# Effect of acupuncture for constipation after ischemic stroke: study protocol for a randomized controlled trial

**DOI:** 10.1186/s13063-018-2750-0

**Published:** 2018-08-22

**Authors:** Tao Zhang, Guiling Wang, Bin Li, Linpeng Wang, Jing Guo, Junxia Hu, Xin Du, Qiuyang Hong, Jingqing Sun, Cunzhi Liu

**Affiliations:** 1 Department of Acupuncture and Moxibustion, Beijing Hospital of Traditional Chinese Medicine affiliated to Capital Medical University, Beijing, China; 2 Department of Acupuncture and Moxibustion, Shunyi Hospital affiliated to Beijing Hospital of Traditional Chinese Medicine, Beijing, China; 3 Dongfang Hospital affiliated to Beijing University of Traditional Chinese Medicine, Beijing, China

**Keywords:** Acupuncture, Constipation, Ischemic stroke, Study protocol

## Abstract

**Background:**

Constipation is a common complication after stroke that can severely influence a patient’s quality of life and rehabilitation. Treatments for constipation after stroke vary. Acupuncture may improve spontaneous bowel movements, quality of life, and clinical symptoms. The study seeks to assess the preliminary effects of acupuncture on constipation after an ischemic stroke.

**Methods/design:**

This is a prospective randomized controlled pilot trial design in which 120 eligible patients will be randomly allocated to one of three groups. The acupuncture group (*n* = 40) will receive acupuncture and routine care, the medication group (*n* = 40) will receive mosapride citrate and routine care, and the control group (*n* = 40) will receive only routine care for ischemic stroke. Patients will be recruited 2 weeks to 6 months after stroke onset and will receive the intervention continuously over 4 weeks, with a follow-up period of 4 additional weeks. Adverse events will be recorded to assess the safety and tolerability of acupuncture for constipation after an ischemic stroke. The primary outcome will be the change in the weekly mean number of complete spontaneous bowel movements. Secondary outcomes will include any change in the weekly mean number of spontaneous bowel movements, mean stool consistency scores, mean straining scores during defecation, and frequency of laxative use. All outcome measures will be assessed at inception, after the intervention (4 weeks), and at the follow-up (8 weeks).

**Discussion:**

This study will provide evidence of the preliminary effects and inform future sample size calculations for studies of acupuncture for constipation following an ischemic stroke. These findings will inform subsequent large-scale randomized controlled trials.

**Trial registration:**

ISRCTN, 22214747. Registered on 17 August 2015.

**Electronic supplementary material:**

The online version of this article (10.1186/s13063-018-2750-0) contains supplementary material, which is available to authorized users.

## Background

Globally, stroke is the second leading cause of mortality and disability among adults over 60 years old [1, 2]. Due to an aging population, dietary changes, pronounced work-related stress, and rapid economic development, there is an increasing prevalence of stroke among younger individuals [3]. Constipation is a common complication after stroke [4]. Population-based studies indicate that approximately 30% to 60% of stroke patients suffer, or have suffered, from constipation following stroke [5–8]. This wide range can likely be attributed to the adoption of different time points [5, 7, 8], the diagnostic criteria used to measure constipation [4, 5] and patient characteristics [5, 6]. Constipation can severely impair a patient’s quality of life and rehabilitation [9]. Consequently, constipation incurs a heavy burden on patients, patients’ families, and society as a whole [10].

Treatments for constipation after stroke vary and include lifestyle changes [11–13], diet changes [14–18], use of laxatives, enemas, prokinetic drugs, and surgically implanted neuro-modulation [19]. Unfortunately, many of these treatments are poorly tolerated or rejected, or there may be a lack of clinical evidence supporting their use. Thus, there is a need for a safe, effective, and non-toxic therapy for constipation after a stroke.

Acupuncture has a history of more than 2000 years in China and plays an important role in traditional Chinese medicine [20]. A recent systematic review indicated that acupuncture is safe and tolerable, and may improve weekly spontaneous bowel movements (SBMs), quality of life, and constipation symptoms [21]. Acupuncture may, therefore, be an effective treatment for post-stroke constipation and it has several advantages compared with other therapies [22]. However, using acupuncture to treat constipation has not been validated with high-quality clinical evidence. In addition, clinical research focusing on acupuncture for constipation after stroke is rare [19]. Therefore, we designed this trial to assess the preliminary effects and inform future sample size calculations for studies of acupuncture for constipation following an ischemic stroke. Our hypothesis is that the effectiveness of acupuncture is no less than that of mosapride and better than routine health care.

## Methods/design

### Study design

**Fig. 1:**
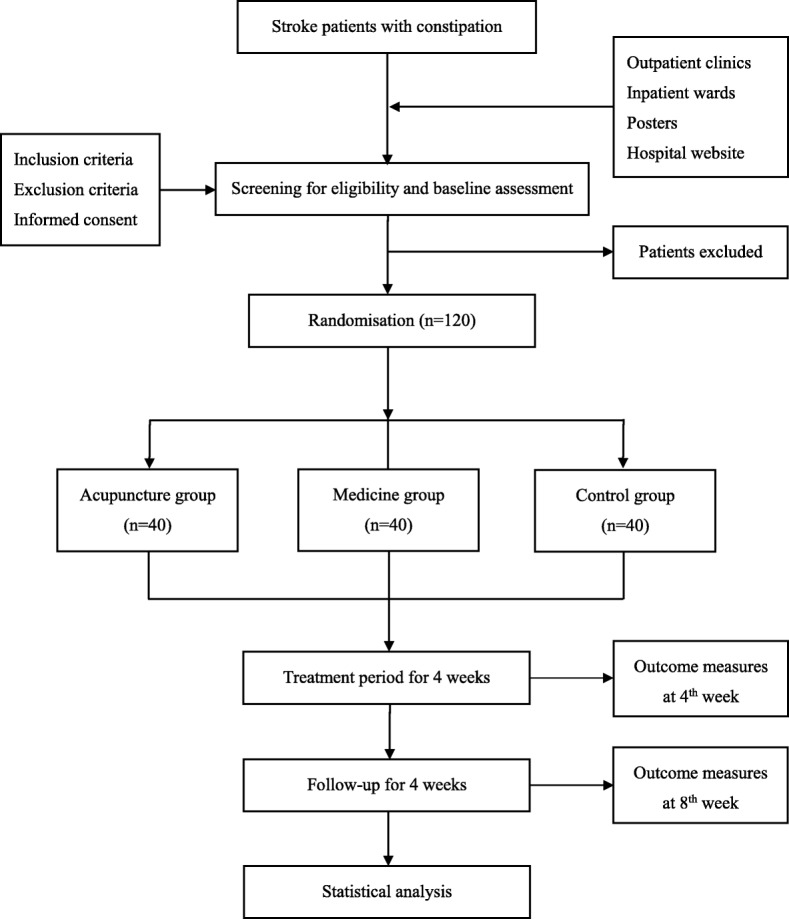
Trial flow chart

This is a prospective parallel-arm randomized controlled pilot trial comparing acupuncture with a pharmacological intervention (mosapride citrate) and usual care (control group). We will recruit patients who have sustained an ischemic stroke and present with constipation, according to predefined inclusion and exclusion criteria, between January 2017 and September 2019. Participants will be randomly allocated to one of three groups and will receive continuous treatment for 4 weeks. Repeat assessments will take place after treatment and later, at 8 weeks after inception. All participants will be asked to keep a defecation diary including weekly number of complete spontaneous bowel movements (CSBMs), SBMs, stool consistency, defecation difficulty, and laxative use and dosage, beginning at baseline during the 8-week assessment period. All outcomes will be assessed at baseline, and at 4- and 8-weeks after randomization, according to the defecation diary (Fig. 1).

### Randomization and allocation concealment

Participants will be randomly assigned to one of three groups (the acupuncture group, the medication group, or the control group) using a 1:1:1 ratio. The generation and allocation of a random sequence for group assignment will be conducted by the Research Centre of Clinical Epidemiology, which is affiliated to Peking University Third Hospital in China. Individuals involved in participant randomization will be blinded and will not participate in the recruitment and inclusion of participants. The block randomization method (block size of 6) will be adopted to generate the random sequence, using the Statistical Analysis System (V.9.1.3; SAS Institute Inc., Cary, North Carolina, USA). Opaque computer-generated sealed envelopes will be produced for the randomization and allocation. The envelopes will be numbered consecutively with a serial number on the outside and will contain the allocation information. An envelope will be opened when a participant enters the study after the baseline measurements have been taken. Participants will be allocated to one of the three groups and receive an intervention according to their group allocation. The random allocation sequence and the opaque sealed envelopes will be kept separately by two specific researchers.

### Blinding

Data managers (including the telephone interviewer during the follow-up period) and statisticians will be blinded to participant group assignment. Therapists, data managers, and statisticians will not be allowed to communicate with others about the participants’ allocation.

### Participants

#### Study population

Patients with ischemic stroke who present with constipation will be recruited from the acupuncture and moxibustion departments of the Beijing Hospital of Traditional Chinese Medicine Affiliated to Capital Medical University in Beijing, China.

#### Sample size

Since there are no previous three-armed randomized controlled trials that investigate this topic, the present study will provide effect size data for sample size calculations of subsequent large-scale randomized controlled trials. For practical reasons, a sample size of 40 per group and a total number of 120 participants will be recruited during the entire study according to outpatient and inpatient censuses of the hospital.

#### Recruitment and baseline assessment

Researchers will recruit participants from outpatient clinics and inpatient wards of the acupuncture and moxibustion departments. Recruitment posters will be posted in the hospital. The posters will contain a brief, Institutional Review Board-approved introduction to the study, the participants, the interventions, and contact information for the researchers. The recruitment information will also be uploaded to the hospital website (http://www.bjzhongyi.com).

Consent will be obtained by one of the investigators, with a witness present. Following written informed consent, eligible patients will be asked to complete a general information form including name, gender, age, medical history, etc. and complete a 1-week defecation diary as the baseline assessment. A neurologist will determine the Glasgow Coma Scale score and the National Institute of Health Stroke Scale score for each participant and these scores will be used to decide whether to include the patient in the study according to the inclusion and exclusion criteria. Personal information about potential and enrolled participants will be collected and maintained confidentially.

#### Inclusion criteria

Inclusion criteria are a diagnosis of ischemic stroke according to the diagnostic criteria specified by the World Health Organization [23], constipation symptoms according to the Rome IV Functional Constipation Criteria [24], 2 weeks to 6 months after stroke onset, aged between 30 and 75 years old, Glasgow Coma Scale score ≥ 7 and National Institute of Health Stroke Scale ≤ 21, no previous medical history of constipation before stroke onset and symptoms of constipation present for at least 2 consecutive weeks after stroke, no use of gastrointestinal drugs 1 week before randomization (except for emergency drugs), no acupuncture treatment for constipation in the previous 2 weeks, and signed informed consent form.

#### Exclusion criteria

Patients with any of the following conditions will be excluded: irritable bowel syndrome or constipation secondary to organic diseases (e.g. endocrine, metabolic, or postoperative diseases); abdominal aneurysms, hepatosplenomegaly, or serious cardiovascular, liver, kidney, or psychiatric diseases; and blood coagulation disorders. Also excluded are pregnant or lactating women and patients requiring administration of anticoagulant agents, such as warfarin or heparin. Participants treated with antiplatelet treatments, such as aspirin or clopidogrel, for the secondary prevention of cerebral infarction are eligible.

### Intervention

The three groups will receive routine health care and secondary prevention as for all other inpatients with ischemic stroke. These treatments include antiplatelet therapies, antihypertensive therapies, glucose control, dyslipidemia control, and dehydration treatments, if necessary. For participants with no SBMs over 4 or more days, use of laxatives (regarded as emergency drugs, such as bisacodyl, glycerin enema etc.) will be permitted as an emergency measure to promote defecation. All information regarding the use of emergency drugs (including date of administration, and laxative types and dosage) will be recorded. Before the initiation of the study, all researchers will receive standardized training regarding the content of the trial, treatment strategies, assessments, and quality control. Interventions will be performed in accordance with the STRICTA [25] and good clinical practice guidelines.

#### Acupuncture group

All the study acupuncturists will be registered practitioners of traditional Chinese medicine with at least 5 years’ clinical experience, as well as a medical education background. The acupuncture treatments and manipulations will be standardized among acupuncture practitioners through live and recorded training. Outpatients will receive treatment in clinics and inpatients will be treated in wards. If inpatients are discharged, they will receive acupuncture in clinics by the same acupuncture practitioner. Sterile needles (size 0.30 × 40 mm and 0.35 × 75 mm; Guizhou Ande Medication Appliance, Ltd., Guiyang City, Guizhou Province, China) will be used.

For the acupuncture, we will use Wang’s classical 10 acupoints formula. These acupoints have been selected and combined for gastrointestinal disease after a stroke based on the clinical experience of an accomplished Chinese acupuncturist, Leting Wang, in the 1960s [26, 27]. The 10 acupoints include CV13 (*shangwan*), CV12 (*zhongwan*), CV10 (*xiawan*), bilateral ST25 (*tianshu*), CV6 (*qihai*), bilateral PC6 (*neiguan*), and bilateral ST36 (*zusanli*). All of the acupoints will be located according to the World Health Organization Standardized Acupuncture Points Location [28]. After sterilizing the skin, needles will be slowly and vertically inserted into ST25 (*tianshu*) bilaterally, without any manipulation. Needle insertion will stop upon piercing of the muscle layer of the abdominal wall (nearly 20 to 60 mm subcutaneously). Known side effects of needle insertion include mild soreness. Other needles will be inserted to a depth of 10–25 mm to predetermined acupoints and manually manipulated by rotation to produce the characteristic sensation of *deqi*. The *deqi* sensation is also called *needle sensation*, and refers to perceived subcutaneous tension around the needle, which is felt by the acupuncture practitioner, and soreness, numbness, distension, and heaviness around the acupuncture point that is felt by the participant. Each session will last for approximately 30 min. All needles will be removed with sterile cotton balls to avoid bleeding.

Each participant will be treated once a day and will receive 16 treatment sessions in total over 4 consecutive weeks. Treatment will be conducted 5 times per week in the first 2 weeks and 3 times per week in the last 2 weeks.

#### Medication group

Participants in the medication group will receive 5 mg of mosapride citrate, 3 times per day for 4 consecutive weeks. Unused tablets will be collected by the researchers at the end of the treatment period.

#### Control group

Participants in the control group will receive routine health care and secondary prevention treatments for ischemic stroke. For participants with no SBMs over 4 or more consecutive days, cathartic treatment will be given. Details related to the usage of cathartic drugs will be recorded during the trial.

#### Follow-up

After the 4-week treatment, all participants will enter an additional 4-week follow-up period. During this time, they will receive routine health care and secondary prevention as provided to all other patients with ischemic stroke. Laxatives will be allowed for use as emergency drugs during the follow-up period. However, acupuncture treatment and mosapride citrate are not permitted during follow-up.

### Outcome measures

#### Primary outcome measure

The primary outcome measure will be the change in the weekly mean number of CSBMs over the 4 weeks of the intervention and the 4 weeks of the follow-up. A CSBM is defined as a SBM unrelated to the administration of laxatives or finger manipulation, accompanied by a self-reported feeling of complete evacuation. Bowel movements achieved with the help of glycerin, enemas, or finger manipulation are not considered spontaneous. If bowel movements occur within 24 h after the intake of laxatives, they will not be considered spontaneous. However, if bowel movements occur more than 24 h after administration of laxatives, they will be considered spontaneous. The total number of CSBMs during the treatment period and follow-up period will be averaged to obtain mean weekly CSBMs.

#### Secondary outcome measures

1. The change in the number of mean SBMs that are unrelated to the administration of laxatives or finger manipulation, with or without a self-reported feeling of complete evacuation. This measure will be calculated in the same way as the CSBMs.
2. The change in mean stool consistency score, as per the Bristol Stool Scale (Table 1). The score will be recorded according to the stool type (e.g. type 1 = 1 point, type 2 = 2 points, etc.) to obtain mean stool consistency scores. The Bristol Stool Scale is a widely used form authored by Dr. K Heaton, reader in medicine, University of Bristol. It can be downloaded from the website https://www.bladderandbowel.org/bowel/bowel-resources/bristol-stool-form-scale/ representations of the different types of stool will be included in the patients’ diaries and serve as visual comparators. A patient will record the type of stool in their diary according to the picture.
3. The change in mean straining scores during defecation. The severity of straining will be graded using a 5-point ordinal scale: 0 = absent, 1 = mild, 2 = moderate, 3 = severe, and 4 = very severe.
4. The change in weekly frequency of laxative use. This includes the total number of times that laxatives are used during the 4-week treatment period and the 4-week follow-up period. Instances of laxative use will be summed and divided by 4 to obtain the weekly frequency during the two periods.

**Table 1:** Bristol Stool Scale

Type	Description
1	Separate hard lumps, like nuts (hard to pass)
2	Sausage-shaped, but lumpy
3	Like a sausage but with cracks on its surface
4	Like a sausage or snake, smooth and soft
5	Soft blobs with clear-cut edges (passed easily)
6	Fluffy pieces with ragged edges, a mushy stool
7	Watery, no solid pieces; entirely liquid

#### Adverse events

Adverse event data will document the occurrence, duration, and severity of adverse reactions (symptoms and signs), and how the event was resolved (or not) during the treatment. For acupuncture, common and expected adverse events include local hematomas; needle breakage; needle retention after treatment; fainting; unbearable prickling, severe pain, or discomfort persisting for more than 1 h after acupuncture; local infections; abscesses; and deterioration of blood laboratory parameters. All participants will receive routine blood, urine, and stool tests; and electrocardiogram, liver function (alanine transaminase and aspartate transaminase), and kidney function tests (serum creatinine and blood urea nitrogen). These tests will be performed twice after randomization and at the end of the 4-week treatment period. The researchers will report all adverse events to the Research Ethics Committee of Beijing Hospital of Traditional Chinese Medicine Affiliated to Capital Medical University and treat the participants with relevant conventional therapy or hospitalization if necessary (the participant’s allocated intervention will be revealed). Researchers will evaluate the relationship between adverse events and the interventions. The number of adverse events will be recorded and divided by the sample size of each group to calculate the incidence.

**Fig. 2:**
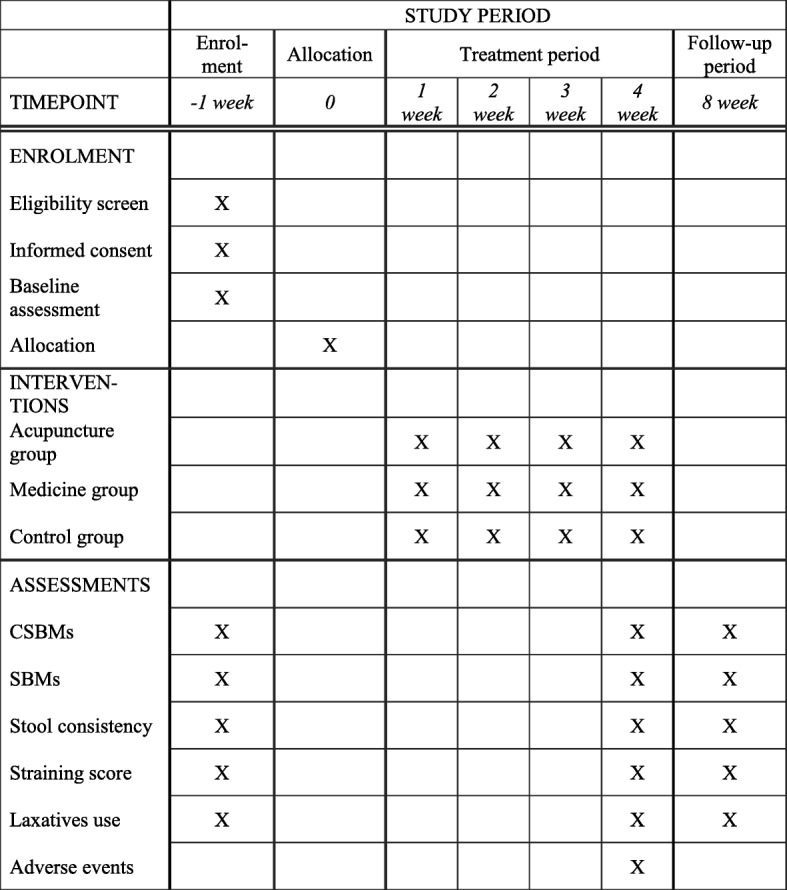
Enrolment schedule, treatment, and outcome measures. CSBM complete spontaneous bowel movement, SBM spontaneous bowel movement

The enrolment schedule, treatment, and outcome measures are presented in Fig. 2.

### Statistical analysis

The analysis of the primary outcome will be based on both a full analysis (intention-to-treat analysis) and per-protocol sets (per-protocol analysis). The analyses of secondary outcomes will be based on the per-protocol sets. The full analysis set refer to the participants who are randomized and receive at least one treatment session. Any missing data will be replaced by the last measured value. The per-protocol sets will be defined as the participants who receive all treatment sessions and completed the study according to the protocol.

The statistical analysis will be performed by a statistician who is blinded to participant group allocation and will not be involved in other aspects of the study. The SPSS statistical package (version 18.0, International Business Machines Corporation, China) will be used to analyze the data. All data will be kept in duplicate by specific researchers. The results of the quantitative data analyses will be presented as means ± standard deviation. The Kolmogorov–Smirnov test will be applied as the normality test for quantitative variables. The Wilcoxon test or analysis of variance (ANOVA) will be used to compare the difference between groups according to the results of the Kolmogorov–Smirnov test. The results for categorical data will be compared with the chi-squared test and presented as a percentage. The statistical significance level will be set at 0.05 (two-sided) with a 95% confidence interval.

### Ethics and dissemination

The study protocol follows the principles of the Declaration of Helsinki and was approved by the Research Ethics Committee of Beijing Hospital of Traditional Chinese Medicine Affiliated to Capital Medical University on 9 July 2015 (reference 2015BL-041-01). The study was registered with ISRCTN as a current controlled trial (22214747). The results will be disseminated through peer-reviewed publications, a master’s thesis, or conference presentations. The data will be anonymized prior to publication to prevent identification of individual participants.

### Monitoring

The study will be regularly monitored by the Data Management and Monitoring Committee of the Good Clinical Practice Department of Beijing Hospital of Traditional Chinese Medicine Affiliated to Capital Medical University. The Data Management and Monitoring Committee operates independently from trial researchers and has no competing interests. The Data Management and Monitoring Committee will monitor the overall quality and completeness of the data, examine original case report forms, interview researchers, verify the record of adverse events, and confirm that the study complies with the principles of this protocol.

## Discussion

Constipation is a common health problem after an ischemic stroke. Relevant research has indicated that constipation after an ischemic stroke can impair the removal of metabolic waste [29], aggravate the development of atherosclerosis, increase the risk of cerebral vasospasm and neural damage, lead to the detachment of deep vein thrombosis and cause thromboembolism, and induce the recurrence of cardiovascular and cerebrovascular diseases [7, 8]. Constipation may severely impair a patient’s rehabilitation and their quality of life. The results of this study will provide preliminary data on the safety, tolerability, and preliminary effectiveness of acupuncture for constipation symptoms after stroke. It will provide evidence for sample size calculations of subsequent large-scale multi-center randomized controlled trials by assessing effect sizes, variability, and the completeness of the data. The study will be a preliminary investigation into the potential enhancement of a currently used intervention and may contribute to changes in future clinical practice.

Acupuncture is popular in China due to its long history, simple administration, therapeutic effects, low cost, and relative safety. It is also increasingly practiced and requested by patients in Western countries as a complementary and alternative medicine [30]. The World Health Organization has drawn up a provisional list of diseases that could potentially be treated with acupuncture, including constipation, stroke, and its sequelae [31, 32]. To treat constipation, we will use Wang’s classical 10 acupoints. These 10 points are combined to treat gastrointestinal disease after a stroke and are based on acupuncture theory and decades of clinical experience [33, 34]. These points were selected because: (1) all 10 acupuncture points have been used for thousands of years in traditional Chinese medicine to treat constipation and other gastrointestinal diseases and (2) from the perspective of evidence-based medicine, clinical randomized controlled trials and systematic reviews also show that these acupuncture points, individually or in combination, may be an effective treatment for constipation [21, 35–38].

The objective of this study is to determine the preliminary effects of acupuncture on constipation after an ischemic stroke. The selection of a control group was a major issue in planning the design of the trial. Reported clinical trials of acupuncture for constipation after a stroke are scarce in both English and Chinese research databases. Consequently, we decided to pursue a pragmatic study design. Due to the scarcity of evidence supporting the use of acupuncture for constipation after a stroke, we did not include a sham acupuncture control group. However, if acupuncture proves to be effective in this study, we will then compare acupuncture with a sham treatment as the next step in clarifying the specific effects of acupuncture. Mosapride, a kind of serotonin 5-hydroxytryptamine 4 (5-HT_4_) receptor agonist, was designated as the positive drug control in the trial. Clinical evidence and guidelines indicate that 5-HT_4_ receptor agonists, such as prucalopride and mosapride, improve the mobility of the gastrointestinal tract and are effective in the management of constipation [39–44]. Since prucalopride is not widely applied in clinical practice in Beijing, we chose mosapride citrate as the drug of choice for the medication group [45, 46].

The study is a prospective randomized controlled pilot trial. Randomization and allocation concealment are applied to avoid selection bias. Blinding is used to minimize performing bias and detection bias. However, methodological limitations still exist in the study. Due to the nature of acupuncture manipulation, the therapists and the participants cannot be blinded in this study. Therapists have to carry out the acupuncture manipulations and control the participant’s sensation of needling. In acupuncture trials, genuine double blinding is difficult to achieve, especially in countries with populations aware of acupuncture (Additional file 1).

## Additional file

Additional File 1: SPIRIT 2013 Checklist: Recommended items to address in a clinical trial protocol and related documents. (DOC 122 kb)

## Abbreviations

5-HT_4_: 5-hydroxytryptamine 4
ANOVA: Analysis of variance
CSBM: Complete spontaneous bowel movement
SBM: Spontaneous bowel movement
